# Impact of Consanguinity and Familial Aggregation on Vitiligo Epidemiology in Saudi Arabia: A Case-Control Study

**DOI:** 10.7759/cureus.63971

**Published:** 2024-07-06

**Authors:** Amr Molla, Raed Jannadi, Abdulfatah Alayoubi, Hamza Domlo, Yazeed Alharbi, Yara Alrehaili

**Affiliations:** 1 Department of Medicine, College of Medicine, Taibah University, Madinah, SAU; 2 Department of Family and Community Medicine and Medical Education, College of Medicine, Taibah University, Madinah, SAU; 3 Department of Basic Medical Sciences, College of Medicine, Taibah University, Madinah, SAU; 4 College of Medicine, Taibah University, Madinah, SAU

**Keywords:** epidemiology, familial aggregation, autoimmune skin disorders, saudi arabia, genetic predisposition, consanguinity, vitiligo

## Abstract

Background

Vitiligo, characterized by depigmented patches due to melanocyte loss, involves genetic, autoimmune, and environmental factors. Recent studies suggest a link between family history, consanguinity, and vitiligo prevalence, particularly in regions with prevalent consanguineous marriages. This study explored the relationship between consanguinity and familial vitiligo prevalence in Saudi Arabia.

Methods

A case-control study enrolled 792 participants from Saudi dermatology clinics (382 vitiligo cases, 408 controls). Family histories and consanguinity levels were assessed. Logistic regression analysis, adjusting for relevant variables, evaluated associations.

Results

Significant associations were found between vitiligo and both parental consanguinity and family history. Cases had higher consanguinity rates, with 246 out of 382 (64.4%), compared to controls, with 161 out of 408 (39.5%). A positive family history of vitiligo was more common in cases, with 184 out of 382 (48.2%) than in controls, with 90 out of 408 (22.1%). Logistic regression identified parental consanguinity and positive family history as significant risk factors for vitiligo, with adjusted odds ratios (aOR) of 2.39 and 2.92, respectively. Their synergistic effect notably amplified the risk (aOR = 7.58), indicating a complex genetic and familial influence on vitiligo in Saudi Arabia.

Conclusions

Consanguinity showed a significant association with vitiligo prevalence, highlighting genetic factors' role. Further genetic research is needed to identify specific mutations in vitiligo among consanguineous populations. Genetic counseling and awareness programs are crucial in regions with high consanguinity rates to mitigate vitiligo and other genetic disorders' risks.

## Introduction

Vitiligo is a chronic autoimmune condition characterized by the loss of skin pigment in macules and patches due to targeted destruction of melanocytes. The exact cause remains unclear, but it is believed to involve alterations in the immune system, genetic predispositions, stress, or exposure to sunlight. Sometimes, vitiligo is associated with other autoimmune disorders, especially thyroid diseases. The condition affects about 1% of the global population, with a typical onset in early adulthood. Available treatments include topical medications, light therapy, surgical interventions, and cosmetic solutions [1-3].

In Saudi Arabia, the prevalence of vitiligo is estimated at 3.5%, which is higher than the global prevalence rate of 0.5-2%. Although a higher incidence in females has been noted, this difference is not statistically significant and may be related to the increased reporting of cosmetic concerns among women. Additionally, a significant portion of the vitiligo cases in Saudi Arabia, 51.6%, are found in individuals below the age of 25, with 19.4% occurring in the 25 to 35 age group. The majority of these cases, 93.5%, are among Saudi Arabian nationals. The mean age of onset for vitiligo is generally in early adulthood, and there is no substantial variation in the age of onset between different genders [4].

Additionally, vitiligo patients in Saudi Arabia experience a high prevalence of major depressive disorder, with significant associations between depression severity and factors such as gender, age, income, and vitiligo type, underscoring the necessity for integrated dermatological and psychological care [5].

Genetic studies estimate vitiligo's heritability at 0.75-0.83, with around 70% of the risk attributed to common genetic variants and 30% to rare variants. Over 50 susceptibility loci have been identified, many of which involve genes regulating immune response, apoptosis, and melanocyte function [6,7]. Moreover, a family history of the condition was observed in approximately 30% of individuals affected globally [8,9]. In Saudi Arabia, where consanguineous marriages are a prevalent and deep-rooted cultural practice, there is an increased occurrence of certain inherited and rare genetic dermatological disorders, which are less common in other countries [10]. Vitiligo is among these conditions that are more frequently observed in the Saudi population.

This study aims to examine the relationship between the incidence of vitiligo in families with consanguineous marriages and the family history of vitiligo among affected individuals. The objectives of our study are to identify the prevalence of familial vitiligo in affected individuals and investigate whether familial vitiligo and consanguineous marriages increase the likelihood of offspring developing vitiligo.

## Materials and methods

Study design and sample

The study was conducted at Taibah University, Madinah, Saudi Arabia. This retrospective case-control study included a total of 790 participants, with 382 patients diagnosed with vitiligo forming the case group and a control group consisting of 408 patients without a vitiligo diagnosis. These patients were selected by a simple random sampling method from participant hospitals in different regions of Saudi Arabia. Patients in the case group were selected from participant hospital database records, covering a five-year period from January 2019 to December 2023.

The inclusion criteria for the study were cases of vitiligo that had to be confirmed by a dermatologist. Participants could be of any age and gender, provided they resided in Saudi Arabia. Additionally, willingness to participate in the study and provide informed consent was required.

The exclusion criteria for the study included patients with other skin depigmentation disorders that are not vitiligo. Additionally, patients with cognitive impairments or conditions that hinder understanding or providing informed consent were excluded.

The control group comprised non-vitiligo patients who had various dermatological infectious diseases (e.g., tinea, molluscum contagiosum, warts, and herpes); additionally, these patients were not previously diagnosed with genetically susceptible dermatoses (e.g., psoriasis, atopic dermatitis, ichthyosis, etc.). This group was sourced from the same healthcare facilities as a case group for a comparative analysis.

Data collection

Data were obtained through direct physician inquiries, phone interviews, self-administered questionnaires, and hospital patient records. Data were collected using a structured questionnaire and divided into two sections. The first section gathered demographic data, including sex, age, and nationality. The second section focused on the family history of vitiligo and parental consanguinity (Appendix 1 and Appendix 2).

Study variables

Independent Variables

Family history of vitiligo: The survey provided a spectrum of response options to ascertain the family history of vitiligo in affected individuals, which were categorized into three groups. The first group consists of first- and second-degree relatives; the second group encompasses other relatives, including third- and fourth-degree relatives and distant kin; and the third group comprises individuals with no family history of vitiligo. We explain the definition of family history degrees.

First-degree relatives were the individuals most closely related to a person. They share approximately 50% of their genes with the individual. First-degree relatives include parents (mother and father), siblings (brothers and sisters), and children (sons and daughters).

Second-degree relatives were those who shared approximately 25% of their genes with the individual. Second-degree relatives include grandparents, grandchildren, aunts, uncles, nephews, nieces, and half-siblings.

Third-degree relatives share roughly 12.5% of their genes with the individual. Third-degree relatives include great-grandparents, great-grandchildren, great-aunts, great-uncles, and first cousins.

Fourth-degree relatives are relatives who share about 6.25% of their genes with the individual. Fourth-degree relatives include great-great-grandparents, great-great-grandchildren, and first cousins once removed (the children of one's first cousins or the first cousins of one's parents).

Distant relatives encompass all other relatives not included in the first four degrees. Distant relatives share less than 6.25% of their genes with the individual. This group includes more distantly related cousins (second cousins, third cousins, etc.) and relatives connected through marriage or adoption that do not share a blood relationship.

Parental consanguinity: The survey inquired about parental consanguinity among affected individuals, offering the following response options: first, second, third, fourth, distant cousin, or no blood relationship between parents. However, in our study, the degrees of consanguinity are categorized into four groups. The first group includes parental consanguinity between first cousins. The second group encompasses consanguineous marriages between second or third cousins. The third group comprises parents who are fourth cousins, other cousins, or distant cousins. The fourth group consists of individuals with no consanguinity between their parents. We explain the consanguinity marriage degrees. 

First cousins refer to parents who are first cousins and share a set of grandparents. That is, each parent is the child of a sibling of the other parent's parent.

Second cousins refer to parents who are second cousins and share a set of great-grandparents. This means that each parent's grandparents are siblings.

Third cousins refer to parents who are third cousins and have a shared set of great-great-grandparents. In this case, each parent's great-grandparents are siblings.

Fourth cousins refer to parents who are fourth cousins and share a set of great-great-great-grandparents, meaning that the great-great-grandparents of each parent are siblings.

Distant cousins refer to any consanguineous relationships beyond the fourth degree, where the shared common ancestor is more distantly related than a great-great-great-grandparent.

Dependent Variable

Vitiligo: In the case group, all patients are diagnosed with vitiligo utilizing the following criteria: the Vitiligo Area Scoring Index (VASI), which reflects the thoroughness of the vitiligo assessments [11], and Wood's lamp examination, which revealed depigmented patches in the form of macules occurring at typical vitiligo sites as per the vitiligo classification. Additionally, other depigmented skin disorders are excluded as part of the diagnostic process.

Covariates

Sex was categorized as male or female. Age was measured in years. However, nationality was divided into Saudi and non-Saudi, considering the small number of non-Saudi patients.

Data analysis

Data were analyzed using IBM SPSS Statistics for Windows, Version 29 (released 2023; IBM Corp., Armonk, New York, United States). A chi-square test of independence was used to assess the association between categorical variables. Logistic regression analysis was conducted to calculate the adjusted odds ratios (aORs). The final regression model was adjusted for age, gender, nationality, parental consanguinity, and family history of vitiligo. A p-value of less than 0.05 was considered statistically significant.

## Results

The demographic characteristics of the study participants are summarized in Table 1. The study included 790 participants: 382 cases (48.1%) and 408 controls (51.9%). On average, cases were about two years younger than controls (29.89 vs. 31.92); however, this difference was statistically insignificant (p=0.07). Gender differences were significant, with females comprising 189 out of 382 (49.5%) of cases against 237 out of 408 (58.1%) in the control group (p=0.015). Nationality-wise, Saudis made up 339 out of 382 (88.7%) of cases versus 387 out of 408 (94.9%) in controls (p=0.002). Moreover, parental consanguinity revealed a significant association with vitiligo, with 246 out of 382 (64.4%) of cases having parents who were cousins, compared to 161 out of 408 (39.5%) in controls (p<0.001). Second or third-cousin parental consanguinity marriages were more frequently affected than other consanguinity degrees, with 121 out of 382 (31.7%) in cases versus 36 out of 408 (8.8%) in controls (p<0.001). Furthermore, a family history of vitiligo was significantly more common in cases, with 184 out of 382 (48.2%) compared to 90 out of 408 (22.1%) in controls (p<0.001). Participants indicated that first- or second-degree relatives with vitiligo were more common in cases, with 77 out of 382 (20.2%) compared to 44 out of 408 (10.8%) in controls (p<0.001), as referred to in Table 1.

**Table 1:** Comparison between case and control groups across study variables Note: P-value of less than 0.05 was considered statistically significant.

Variables		Total N (%)	Case N (%)	Control N (%)	p-value
		790 (100)	382 (48.1)	408 (51.8)	
Age, years	Mean (SD)	30.94 (15.78)	29.89 (17.99)	31.92 (13.33)	.07
Gender	Male	364 (46.1)	193 (50.5)	171 (41.9)	.015
	Female	426 (53.9)	189 (49.5)	237 (58.1)	
Nationality	Saudi	726 (91.9)	339 (88.7)	387 (94.9)	.002
	Non-Saudi	64 (8.1)	43 (11.3)	21 (5.1)	
Parental consanguinity	Yes	407 (51.5)	246 (64.4)	161 (39.5)	p< .001
	No	383 (48.5)	136 (35.6)	247 (60.5)	
Consanguinity degrees	1^st^ cousins	118 (14.9)	59 (15.4)	59 (14.5)	p< .001
	2^nd^ / 3^rd^ cousins	157 (19.9)	121 (31.7)	36 (8.8)	
	Other / distant cousins	132 (16.7)	66 (17.3)	66 (16.2)	
Family history of vitiligo	Yes	274 (34.7)	184 (48.2)	90 (22.1)	p< .001
	No	516 (65.3)	198 (51.8)	318 (77.9)	
Family history degrees	1^st^ / 2^nd^ degree relatives	121 (15.3)	77 (20.2)	44 (10.8)	p< .001
	Other degrees / distant relative	153 (19.4)	107 (28)	46 (11.3)	

Logistic regression analysis revealed that individuals with parental consanguinity have a significantly higher risk of developing vitiligo, with an adjusted odds ratio (aOR) of 2.39 (95% CI: 1.76 - 3.24, p<0.001). Additionally, the risk increases with an aOR of 1.57 (95% CI: 1.01 - 2.44, p=0.044), indicating a marginally significant elevation in vitiligo risk for individuals whose parents are first cousins compared to non-consanguineous parents. A more substantial risk increase is observed with second or third-cousin parents, with an aOR of 5.13 (95% CI: 3.29 - 7.98). Having a family history of vitiligo also significantly increases the vitiligo risk, with an aOR of 2.92 (95% CI: 2.11 - 4.03, p<0.001). Furthermore, reporting a vitiligo case in first- or second-degree relatives implies a significant risk increase with an aOR of 2.73 (95% CI: 1.76 - 4.23, p<0.001). The risk remains high when having a distant relative with vitiligo (aOR = 2.95, 95% CI: 1.96 - 4.45), suggesting that even distant familial connections to vitiligo significantly affect an individual's risk of developing vitiligo (Table 2).

**Table 2:** Logistic regression analysis of familial variables on the risk of vitiligo development ^a^ aOR was calculated by including age, gender, nationality, parental consanguinity and family history of vitiligo in the final regression model. Note: P-value of less than 0.05 was considered statistically significant. aOR: adjusted odds ratios; CI: confidence interval

Variables		aOR^a^ (95% CI)	p-value
Parental consanguinity	No	1	
	Yes	2.39 (1.76 – 3.24)	p< .001
Consanguinity degrees	No consanguinity	1	
	1^st^ cousins	1.57 (1.01 – 2.44)	.044
	2^nd^ / 3^rd^ cousins	5.13 (3.29 – 7.98)	p< .001
	Other / distant cousins	1.59 (1.04 – 2.42)	.03
Family history of vitiligo	No	1	
	Yes	2.92 (2.11 – 4.03)	p< .001
Family history degrees	No family history	1	
	1^st^ / 2^nd^ degree relatives	2.73 (1.76 – 4.23)	p< .001
	Otherdegrees / distant relatives	2.95 (1.96 – 4.45)	p< .001

Table 3 illustrates the effect of the interaction between parental consanguinity and a positive family history of vitiligo on the risk of developing vitiligo. The group with no family history of vitiligo and no parental consanguinity serves as the reference group with an aOR of 1, indicating the baseline risk of developing vitiligo without the influence of consanguinity or a family history. The table demonstrates that parental consanguinity alone can elevate the risk of vitiligo, even in the absence of a family history, with an aOR of 1.93 (95% CI: 1.34 - 2.78, p<0.001). Similarly, having a family history of vitiligo without parental consanguinity also increases the risk, with an aOR of 1.99 (95% CI: 1.23 - 3.25, p=0.005). The combination of both a positive family history and parental consanguinity significantly heightens the risk of vitiligo, with an aOR of 7.58 (95% CI: 4.93 - 11.66, p<0.001).

**Table 3:** Interactive effects of parental consanguinity and familial vitiligo on the risk prediction for vitiligo development ^a^ aOR was calculated by including age, gender, and nationality in the final regression model. Note: P-value of less than 0.05 was considered statistically significant. aOR: adjusted odds ratios; CI: confidence interval

Variables	aOR^a^ (95% CI)	p-value
No family history & no consanguinity	1	
No family history & positive consanguinity	1.93 (1.34 – 2.78)	p< .001
Positive family history & no consanguinity	1.99 (1.23 – 3.25)	.005
Positive family history & positive consanguinity	7.58 (4.93 – 11.66)	p< .001

## Discussion

The findings of this research demonstrate a significant correlation between parental consanguinity and the occurrence of vitiligo, revealing that progeny from consanguineous marriages possess a more than twofold increased likelihood of vitiligo manifestation relative to offspring of non-consanguineous parentage. Furthermore, individuals with a familial history of vitiligo exhibit nearly a threefold augmented risk of disease acquisition in comparison to counterparts devoid of such a history. Notably, the investigation elucidates a synergistic amplification when both factors coexist, markedly exceeding the sum of their individual effects. This synergy underscores the intricate contribution of genetic predisposition and familial lineage to vitiligo pathogenesis. These outcomes are congruent with the research's aim to delineate the genetic and familial influence on vitiligo within the Saudi Arabian demographic, thereby enhancing the scientific understanding of its epidemiology.

Moreover, this study has identified that the prevalence of a family history of vitiligo among the case group is significantly higher at 48.2% compared to the reported literature, where only 30% of vitiligo patients have a positive family history. This disparity underscores the unique genetic and environmental interactions potentially influencing vitiligo incidence in the Saudi Arabian population, further emphasizing the importance of considering regional specificities in genetic research related to vitiligo [8,9,12].

The study's findings reveal a pronounced genetic predisposition to vitiligo, significantly influenced by both consanguinity and familial aggregation to vitiligo in Saudi Arabia. Its detailed demographic and genetic analysis, unprecedented in scope with such a large sample size, provides valuable insights into the disease's etiology within a specific cultural context. This investigation uniquely correlates the high-risk incidence of vitiligo with consanguineous marriages, a connection not previously established with this degree of specificity.

In addition, this observation underscores the role of autosomal recessive loci, more commonly expressed in consanguineous populations, in elevating susceptibility to vitiligo development. Recent research highlights the complex genetic basis of vitiligo, involving multiple susceptibility loci and genetic heterogeneity. Genome-wide scans and candidate gene studies have identified several genes linked to melanin biosynthesis, the oxidative stress response, and immune regulation [13,14]. Additionally, the strong link between vitiligo and family medical history further reinforces the genetic contribution of the disease, illustrating the intricate interplay between genetic factors and familial patterns in the manifestation of vitiligo. Our study uniquely focuses on the Saudi population, characterized by a high prevalence of consanguinity, which may enhance the expression of recessive and codominant genetic factors [15,16]. The prevalence of vitiligo in Saudi Arabia is notably higher at 3.5% compared to the global average of 1%, highlighting the need to explore unique genetic and environmental factors. Another study found a significant association between parental consanguinity, particularly first-cousin marriages, and the increased risk of childhood-onset vitiligo. This finding supports the strong genetic component observed in our study, corroborating our results [17]. Moreover, studies in the Qassim and Arar regions of Saudi Arabia have shown that vitiligo heritability is significantly influenced by consanguineous marriages, reinforcing our findings of the genetic predisposition in consanguineous populations [18,19].

This novelty underscores the need for targeted genetic counseling and further genetic research in populations with high inbreeding rates to better understand and mitigate the risk of vitiligo. However, limitations such as self-report bias and the specific cultural settings may lead to the potential misclassification of parental consanguinity and family history degrees, underscoring the need for cautious interpretation of the findings.

The generalizability of the study's findings is best suited to populations engaging in similar socio-cultural behaviors, especially those with a high prevalence of consanguineous marriages. The notable disparities in consanguinity rates between cases and controls hint at a variable genetic predisposition based on regional demographics, potentially affecting genetic counseling and disease management strategies. Although the insights gained shed light on the genetic and familial aspects of vitiligo within the context of Saudi Arabia, caution should be exercised when considering these findings for populations with diverse genetic makeups, cultural norms, and consanguinity practices.

By acknowledging the strengths and limitations of the current study, there's a highlighted need for further exploration across varied cohorts. Investigating vitiligo's progression in affected individuals, alongside the psychological and social effects within consanguineous families, promises a holistic view of its impact. This approach aims for more effective care strategies, underscoring the importance of longitudinal studies to understand the genetic, environmental, and psychosocial dynamics of vitiligo.

## Conclusions

The study suggests that genetic factors related to family history and parental consanguinity independently contribute to the pathogenesis of vitiligo. The elevated familial incidence of vitiligo compared to global averages points to a distinct genetic and environmental interplay. Limitations such as recall bias and regional specificity suggest caution in generalizing findings. Future research is imperative to uncover specific genetic markers and assess vitiligo's psychosocial impacts, aiming to refine genetic counseling and disease management. This study lays a critical foundation for understanding vitiligo's genetic underpinnings, emphasizing the need for comprehensive, culturally sensitive research approaches.

## Appendices

Appendix 1

**Table 4:** Consent and questionnaire form

موافقة خطية (لدراسة تتضمن الكائنات البشرية)					Informed Consent (Studies involving human beings)					
عنوان الدراسة		العلاقة بين البهاق والتاريخ الطبي للعائلة وزواج الأقارب في المملكة العربية السعودية.		The relationship between vitiligo, family medical history, and consanguinity marriage in Saudi Arabia						Title
الهدف العام		تهدف هذه الدراسة إلى تقدير الارتباط بين البهاق والتاريخ الطبي في العائلة وعلاقتها بزواج الأقارب في المملكة العربية السعودية. جميع المعلومات الواردة في هذا الاستبيان سرية تمامًا ولن تتم مشاركتها مع أي طرف ثالث .. سيستغرق الأمر حوالي 5-7 دقائق		This study aims to estimate the relationship between vitiligo and medical history in the family and its relationship to consanguinity marriage in the Kingdom of Saudi Arabia. All information in this questionnaire is totally confidential and won’t be shared with any third parties. It will take you approximately 5-7 minutes.						General Aim
الباحث الرئيسي: د/ عمرو ملا					Principal investigator: Dr. Amr Molla					
الباحثون المساعدون					Co-investigator: (add more rows for other investigators)					
Hamza Alayoubi, Yazeed Alharbi, Yara Alrehaili, Hamza Domlo, Dr. Abdulafatah Alayoubi										
بعد مناقشة هذه الدراسة مع:					Having discussed this research project with:					
الباحث:	عمرو أحمد ملا			Amr Molla					Researcher:	
وقرأت او قرأت لي بلغتي الاولي تفاصيل الدراسة وأوافق طوعا على المشاركة في هذه الدراسة.					I have or have been read to me in my first language, the details of the study explained to me, I agree voluntarily to participate in this study.					
اسم المشارك							Participant's name:			
ولي الأمر (ان كان المشارك في الدراسة اصغر من 18)							Next of kin (if not the participant):			
أفهم أنني سأشارك في دراسة، والتي قد تفيدني أو لا تفيدني بشكل مباشر، لكنني سأقدم معرفة جديدة، والتي يمكن أن تفيد المرضى الآخرين (الأشخاص) الذين يعانون من ظروف مشابهة لظروفي في المستقبل. أفهم أيضًا أن لدي الحق في الانسحاب من هذه الدراسة في أي وقت، وذلك بإخبار الباحث.					I understand that I will be participating in a study, which may or may not benefit me directly, but I will provide new knowledge that could benefit other patients (people) with similar conditions in the future. I also understand that I do have the right to withdraw from this study at any time by telling the researcher.					
التوقيع								Signature		
الباحث						Researcher				

Appendix 2 

**Table 5:** Questionnaire - الاستبيان

What is your age?	ما هو عمر المريض؟
What is your gender?	ما هو جنسه؟
What is your nationality?	ما هي جنسيته؟
Do you have a family history of vitiligo? If yes, please specify the degree of the affected relatives (select all that apply): 1-First-degree relatives (parents, siblings, children) 2-Second-degree relatives (grandparents, grandchildren, aunts, uncles, nephews, nieces, half-siblings) 3-Third-degree relatives (great-grandparents, great-grandchildren, great-aunts, great-uncles, first cousins) 4-Fourth-degree relatives or more distant (great-great-grandparents, great-great-grandchildren, second cousins, or more distant relatives) 5- No family history of vitiligo	هل يوجد تاريخ عائلي للبهاق؟ يرجى اختيار الدرجة إذا كان هناك تاريخ عائلي: ١-الدرجة الأولى: (والد/ة، ابن، اخ، اخت) ٢-الدرجة الثانية: (خال/ة، عم/ة، ابن اخ، ابن اخت) ٣-الدرجة الثالثة: (ابن خال، ابن عم، ابن خالة، ابن عمة) ٤-الدرجة الرابعة: (من نفس القبيلة او العائلة) ٥- لا يوجد تاريخ عائلي
Are your parents related by blood? If yes, please specify the degree of consanguinity between your parents: 1-First cousins 2-Second cousins 3-Third cousins 4-Fourth cousins or more distant 5-No Consanguinity	هل يوجد صلة قرابة بين الام والاب للمصاب بالبهاق؟ يرجى اختيار الدرجة او لا يوجد ١-ابناء العمومة من الدرجة الأولى ٢-ابناء عمومة من الدرجة الثانية ٣-ابناء عمومة من الدرجة الثالثة ٤-ابناء عمومة من نفس القبيلة او العائلة. ٥-لا يوجد صلة قرابة
